# Supplementary material for: Allosteric Effect of Nanobody Binding on Ligand-Specific Active States of the β2 Adrenergic Receptor
Source: J Chem Inf Model. 2021 Nov 15;61(12):6024–37. doi: 10.1021/acs.jcim.1c00826 (PMC8715506; doi:10.1021/acs.jcim.1c00826)
Supplement: Supplementary file 1 — ci1c00826_si_001.pdf [file ci1c00826_si_001.pdf]

Supporting information：

Allosteric effect of nanobody binding on ligand-specific active states of the β2-Adrenergic Receptor

Yue Chen[1], Oliver Fleetwood[1], Sergio Pérez-Conesa[1], Lucie Delemotte[1*]

[1]Science for Life Laboratory, Department of Applied Physics, KTH Royal Institute of Technology, SE-17121 Solna, Sweden.

*Corresponding author, E-mail: lucied@kth.se

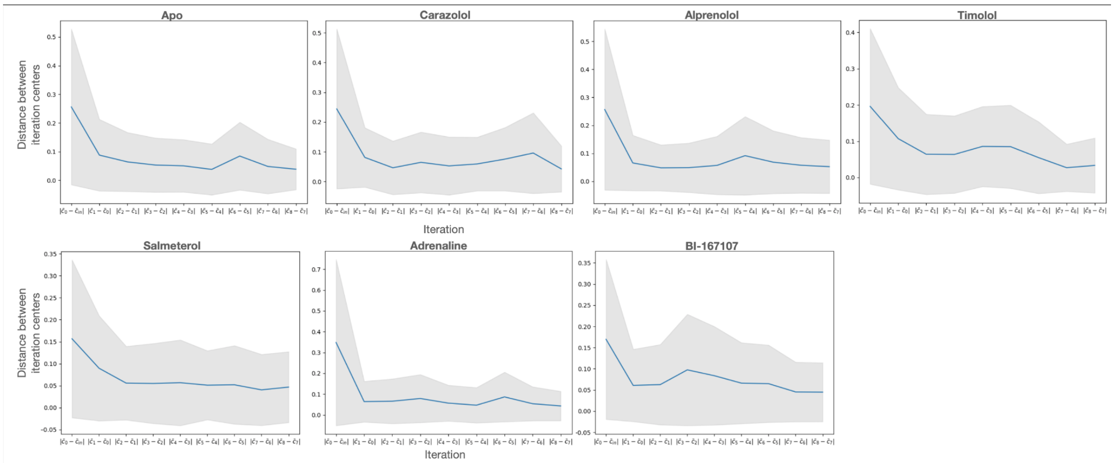

Figure S1. Distance between the center points of replicas over iterations. All systems evolved for 8 iterations, at which point the center points did not drift between iterations. Several systems converged in fewer iterations, but the total simulation time per system was kept fixed for the sake of consistency. Shaded areas represent the standard error of the replicas' distance to the center points.

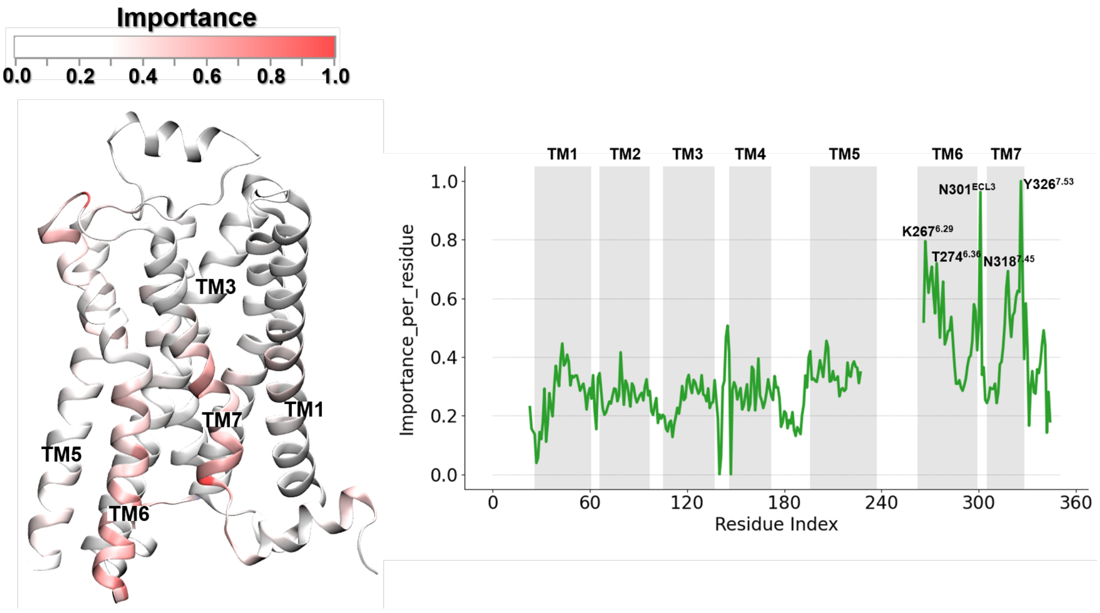

Figure S2. Important residues for discriminating Nb80-bound and -unbound ensembles, derived from PCA on equilibrated active-like ensembles.

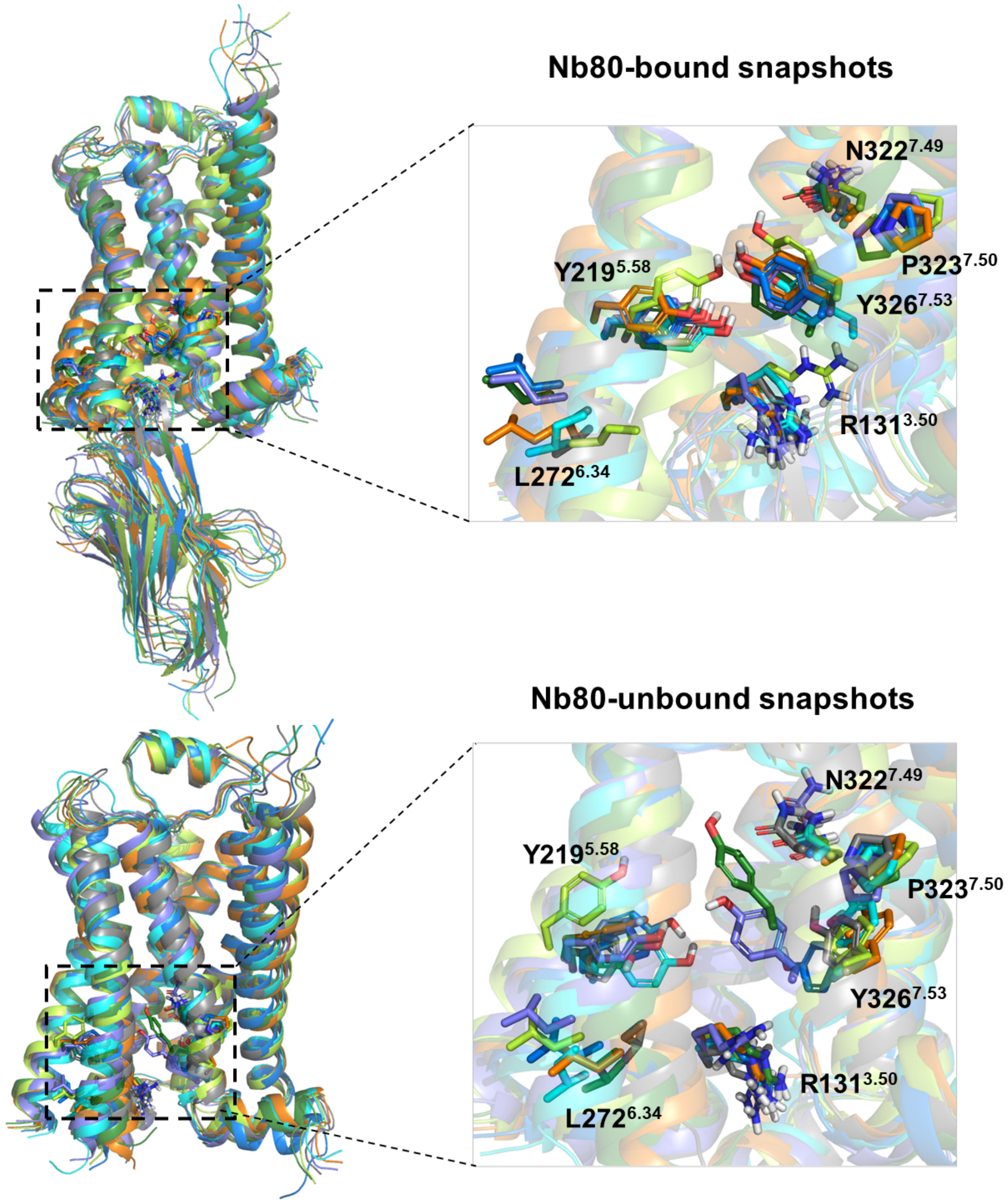

Figure S3. Overlay of representative snapshots of all Nb80-bound and unbound states in the left panel. Rearrangement of residues R131$^{3.50}$, Y219$^{5.58}$, L272$^{6.34}$, N322$^{7.49}$, P323$^{7.50}$ and Y326$^{7.53}$ in the right panel. Apo (dim gray), BI167107 (yellow green), adrenaline (orange), salmeterol (sea green), carazolol (aqua), alprenolol (dodger blue), timolol (slate blue).

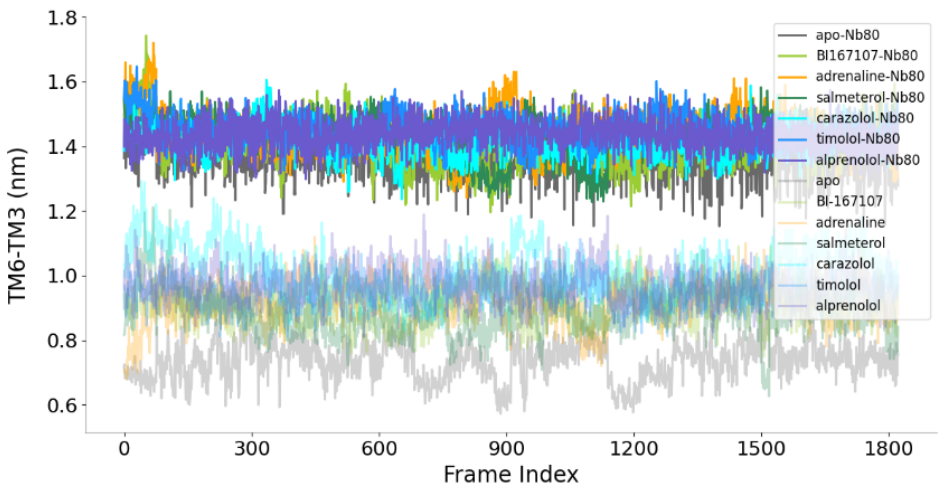

Figure S4. TM6-TM3 distance in all Nb80-bound and unbound ensembles along the MD trajectory.

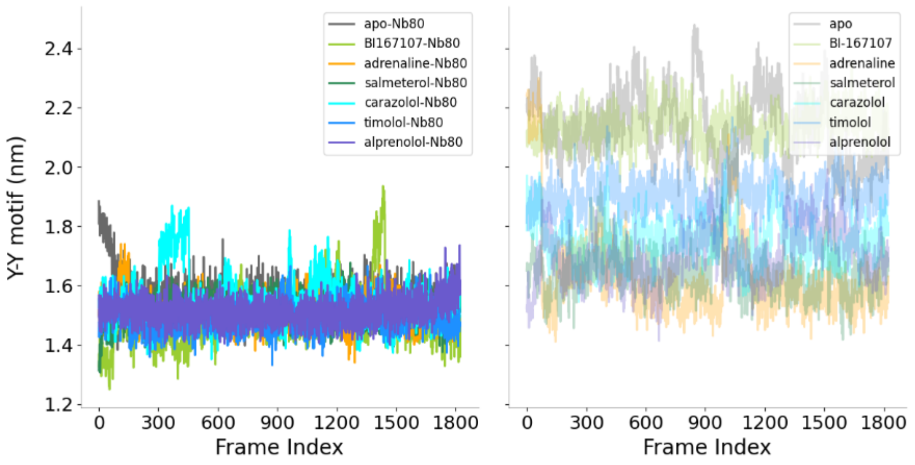

Figure S5. Y-Y motif distance in all Nb80-bound and unbound ensembles along the MD trajectory.

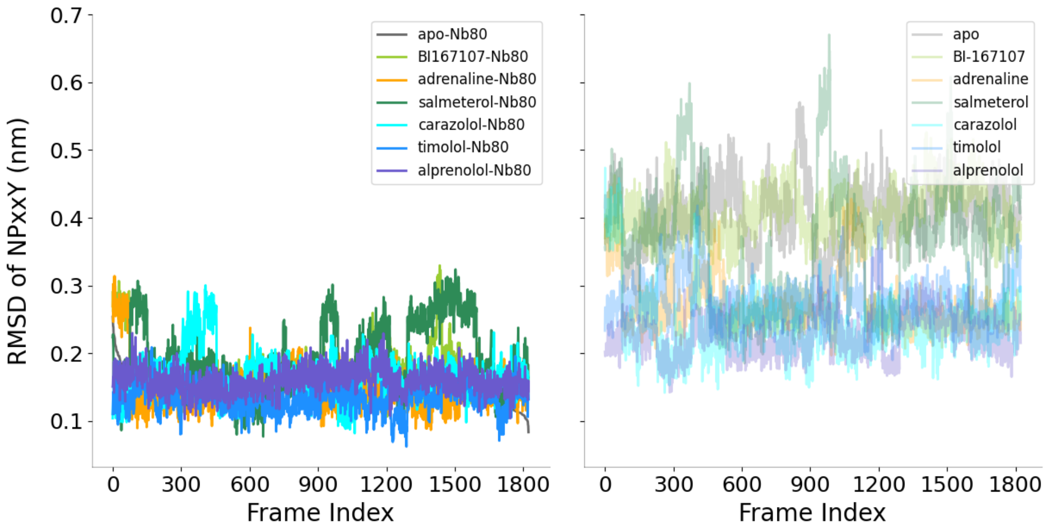

Figure S6. NPxxY motif rmsd in all Nb80-bound and unbound ensembles along the MD trajectory.

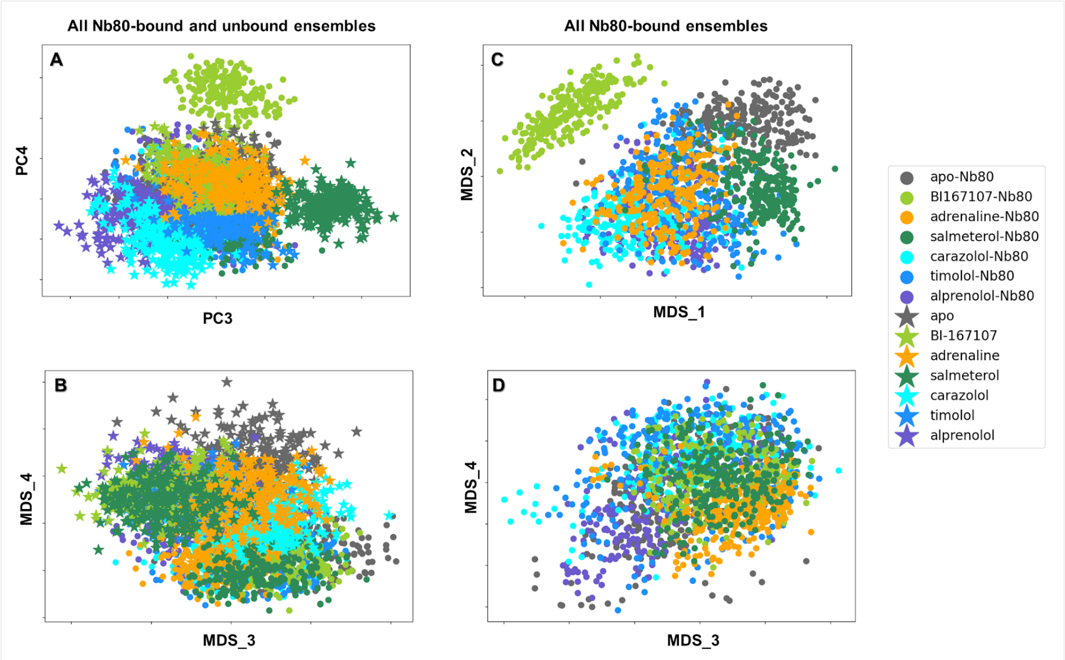

Figure S7. Dimensionality reduction analysis applied to the active-like simulation ensembles. Each point represents a simulation snapshot and is colored according to the ligand and Nb80 bound ensembles. (A-B) PCA and MDS projection onto the third and fourth components of all trajectories with and without Nb80. (C-D) MDS projection on the first four components of trajectories with Nb80.

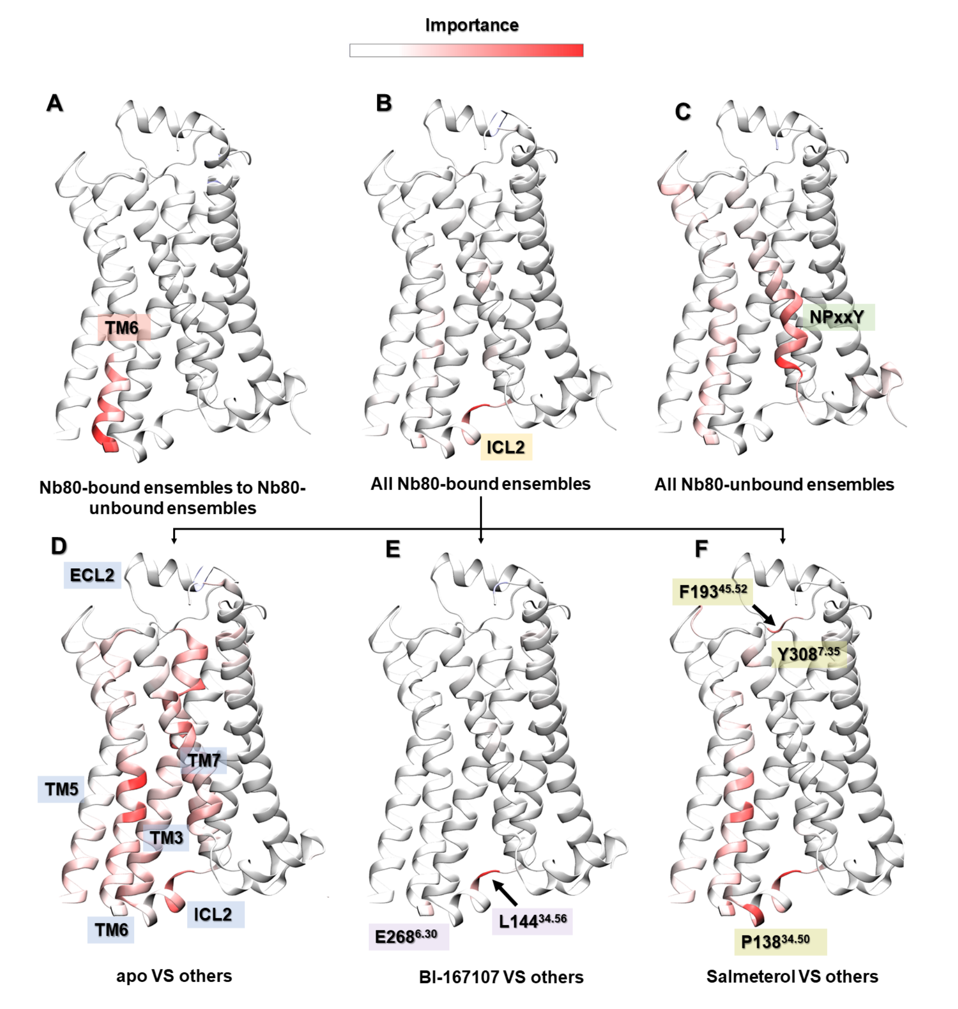

Figure S8. Important residues derived from the equilibrated active-like ensembles for discriminating Nb80- and ligand-dependent activation mechanisms by computing Kullback-Leibler divergence (KL).

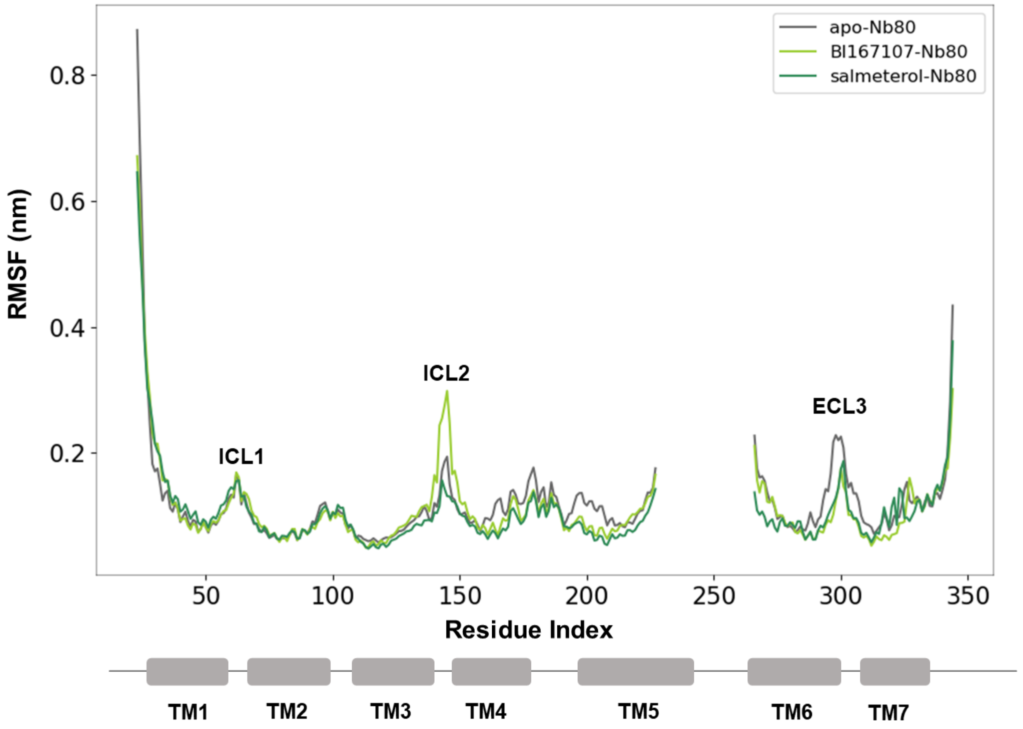

Figure S9. Residue average fluctuations measured as root-mean-square fluctuation (RMSF) in the active-like simulation ensembles of apo, BI167107- and salmeterol-bound β2AR-Nb80 structures.

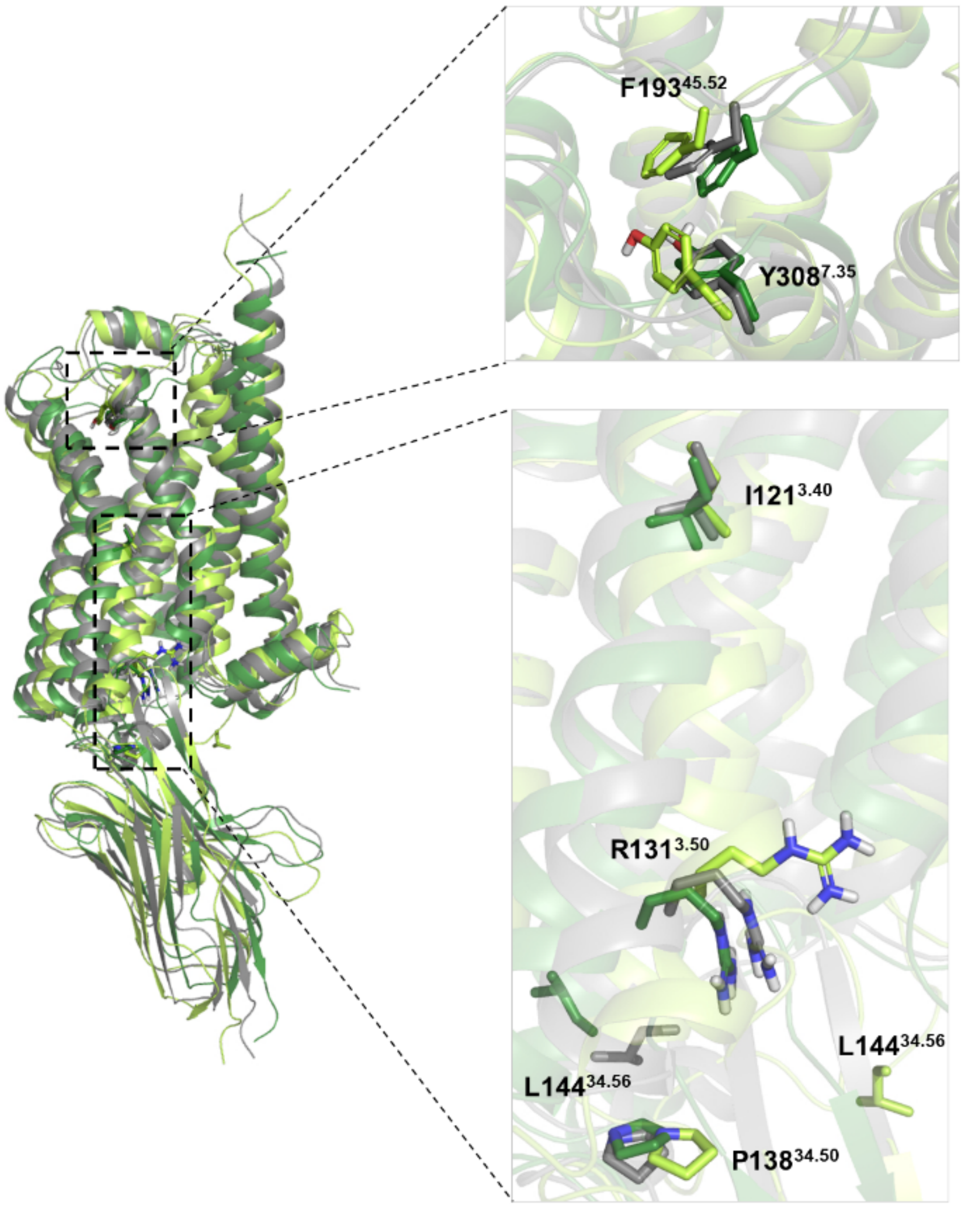

Figure S10. Superimposition of representative snapshots from the apo (dim gray), BI167107- (yellow green) and salmeterol-bound (sea green) β2AR with Nb80. Rearrangement of residues F1934$^{5.52}$, Y308$^{7.35}$, P1383$^{4.50}$, I121$^{3.40}$ and L1443$^{4.56}$, R131$^{3.50}$ are depicted in the right panel.

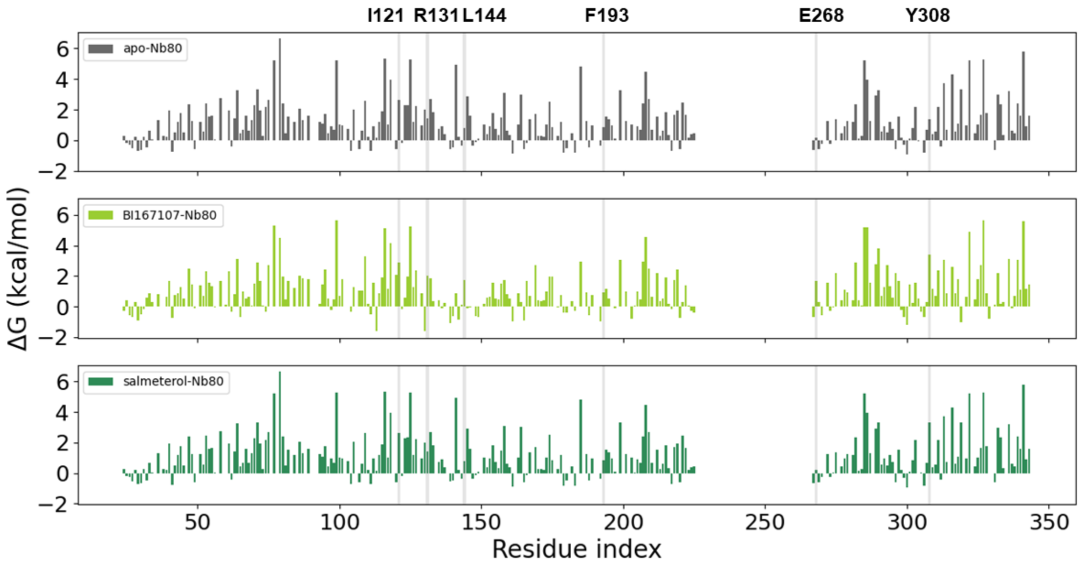

Figure S11. Computational alanine scanning of β2AR residues in apo-, BI167107- and salmeterol-β2AR-Nb80 systems. ΔG shows the predicted stabilizing or destabilizing effect of the alanine mutation on the mutated protein.

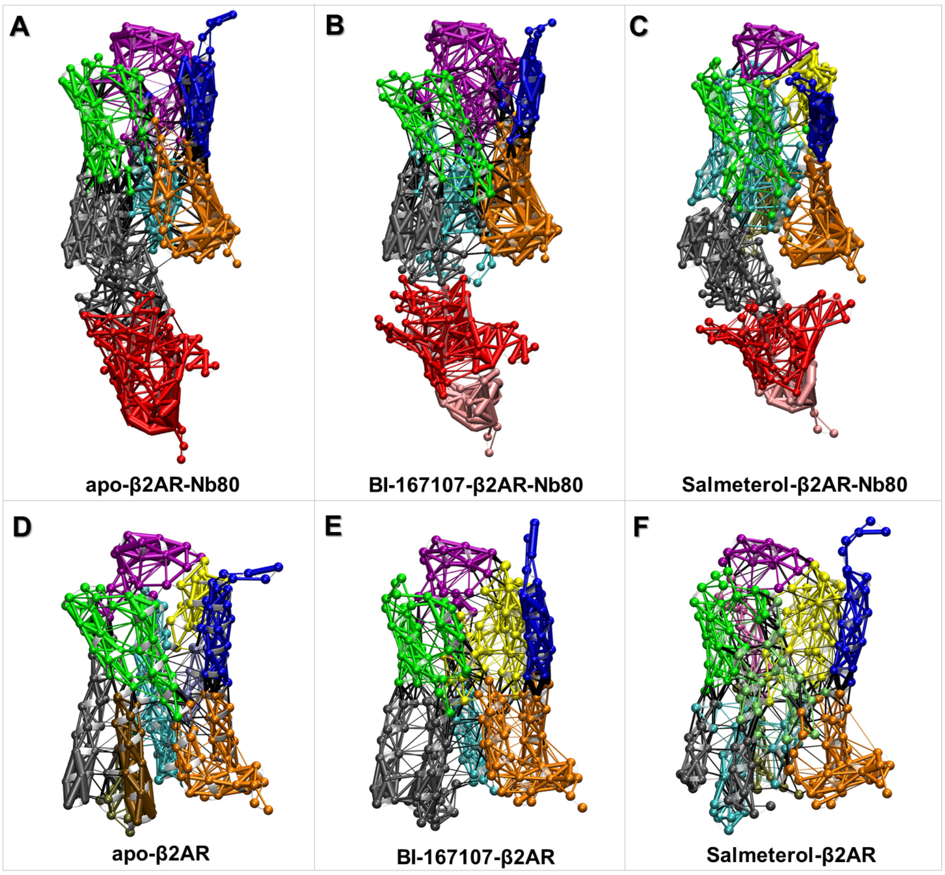

Figure S12. Dynamic networks are identified in the apo, BI167107- and salmeterol-bound β2AR with and without Nb80 bound through community network analysis. (A-C) 3D networks of unliganded, BI167107- and salmeterol-bound forms with Nb80. (D-F) 3D networks of unliganded, BI167107- and salmeterol-bound forms without Nb80. Network communities are colored separately by their ID number. Residues are rendered as spheres and the connecting edges are represented by lines with their width weighted by betweenness.

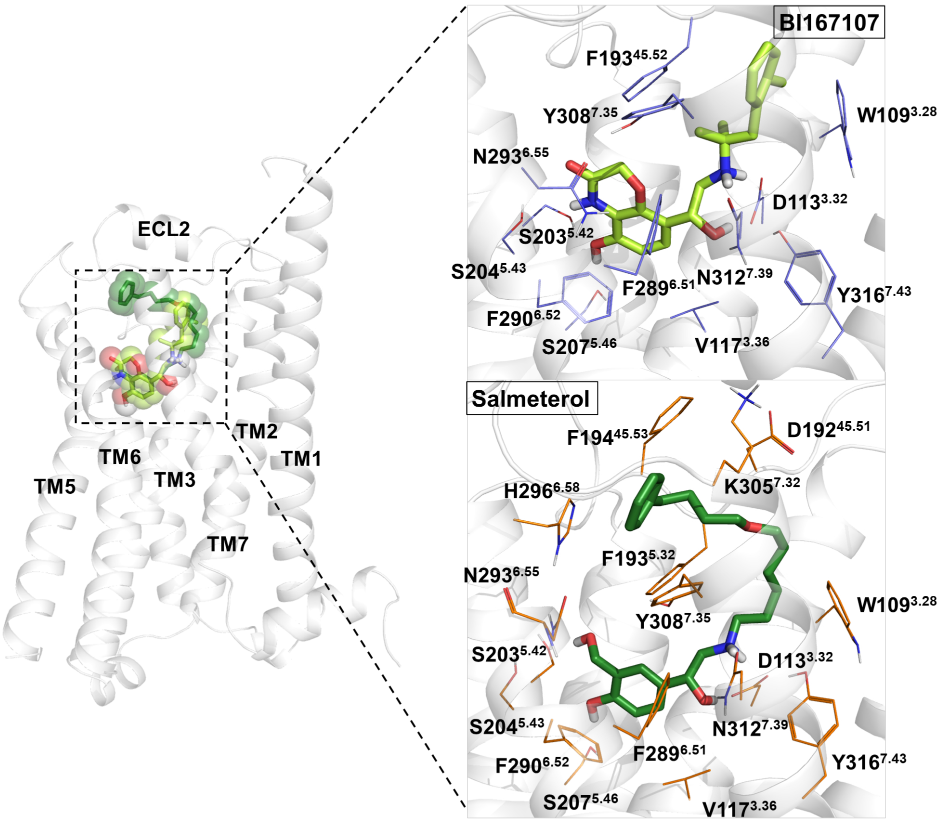

Figure S13. BI167107- (lime) and salmeterol-(dark green) ligand binding pocket of DRD2 (left). BI167107- and salmeterol-binding interactions at a 4.0 Å cut-off (right).

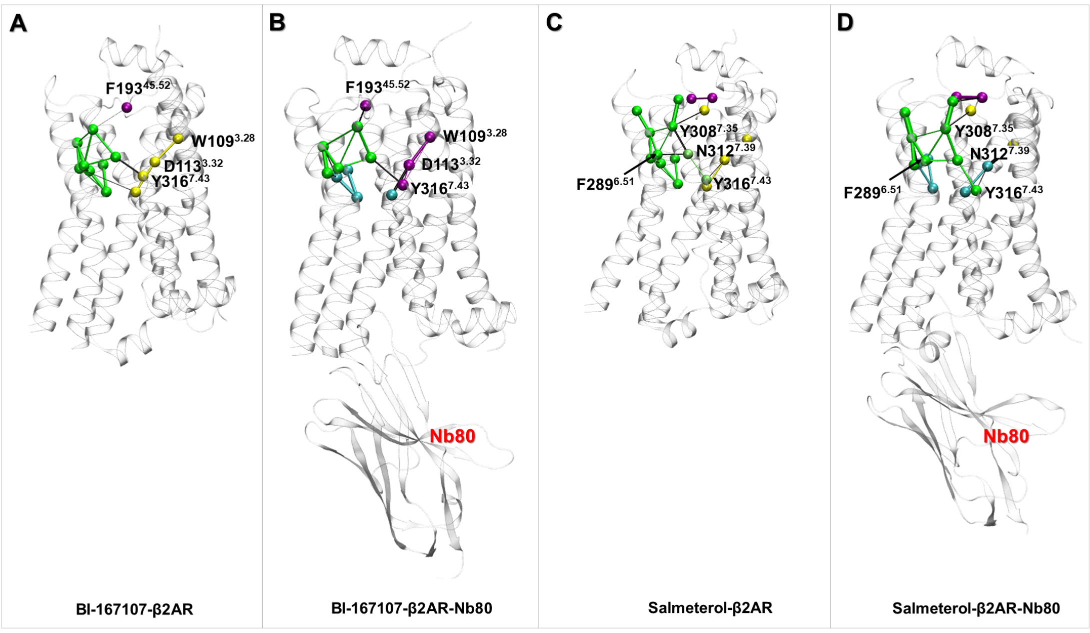

Figure S14. BI167107- and salmeterol-binding pockets with and without Nb80 are highlighted in the dynamic networks through community network analysis. (A-B) 3D networks of BI167107-bound receptor without and with Nb80. (C-D) 3D networks of salmeterol-bound receptor without and with Nb80. Residues are rendered as spheres and the connecting edges are represented by lines with their width weighted by betweenness. The residue color is consistent with that in Figure S12.

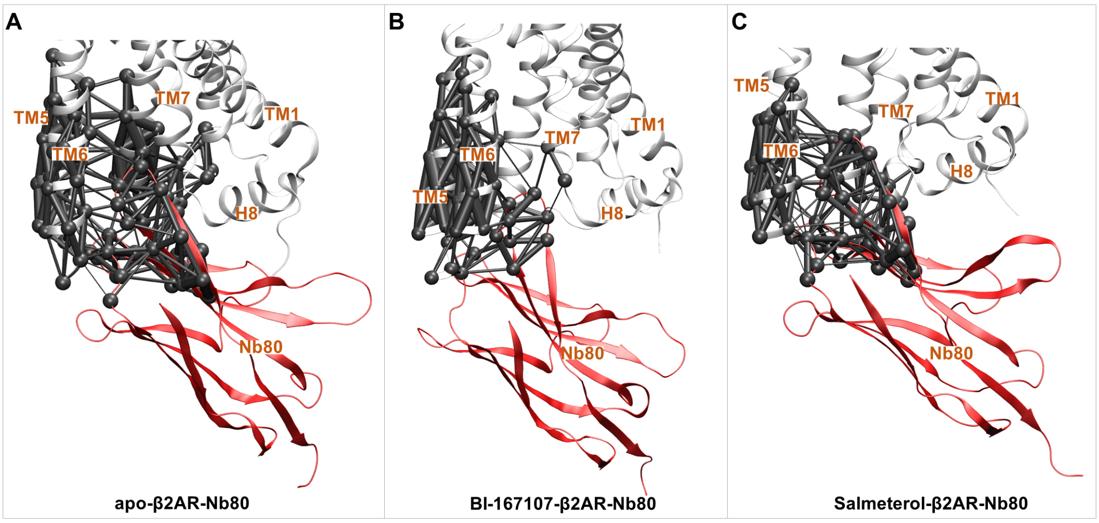

Figure S15. Local network communities involve Nb80 and the intracellular domain of β2AR. β2AR and Nb80 are represented by white and red cartoons respectively. Gray community in the BI167107-β2AR-Nb80 system contains less residues of Nb80 than those in apo and salmeterol-β2AR-Nb80 systems. Residues in the network are rendered as spheres, and the connecting edges are represented by lines with their width weighted by betweenness.
